# Vulnerability of locus coeruleus connections to aging and Alzheimer's disease

**DOI:** 10.1002/alz.71737

**Published:** 2026-08-12

**Authors:** Blanca Zufiria‐Gerbolés, Daniel Vereb, Mite Mijalkov, Massimiliano Passaretti, Giovanni Volpe, Zhilei Xu, Thomas Hinault, Sara Garcia‐Ptacek, Joana B. Pereira, for the Alzheimer's Disease Neuroimaging Initiative

**Affiliations:** ^1^ Department of Clinical Neuroscience, Division of Neuro Karolinska Institutet Stockholm Sweden; ^2^ Department of Human Neurosciences Sapienza University of Rome Rome Italy; ^3^ Department of Physics Goteborg University Goteborg Sweden; ^4^ UNICAEN PSL Université Paris Paris France; ^5^ Department of Neurobiology Care Sciences and Society Karolinska Institutet Stockholm Sweden

**Keywords:** aging, Alzheimer's disease, brain connectivity, locus coeruleus, neuromodulatory nuclei, Tau pathology

## Abstract

**INTRODUCTION:**

The locus coeruleus (LC) is important in coordinating communication between brain regions through its widespread connections. However, the organization of its connections, and its changes with aging and Alzheimer's disease (AD), remains unclear.

**METHODS:**

We mapped whole‐brain white matter connections from the LC in two independent cohorts: one spanning the adult lifespan and another covering the AD continuum.

**RESULTS:**

We identified a novel dorsal–ventral organization of LC connectivity that showed changes common to aging and AD or specific to AD, was linked to gene expression patterns, and was associated with cognitive performance and tau pathology in the entorhinal cortex. We also developed an imaging marker, LC gap, capturing LC connectivity deviations associated with slower tau accumulation in cognitively normal individuals at high risk for AD.

**DISCUSSION:**

These findings provide new insights into LC connectivity in aging and AD, highlighting its potential role as a resilience marker against AD neurodegeneration.

## Highlights

- For the first time, we mapped the whole‐brain white matter connections from the Locus Coeruleus (LC) to the rest of the brain across aging and Alzheimer's disease (AD) continuum.
- Revealed a novel dorsal–ventral organization LC connectivity that presents changes common to aging and AD or specific to AD.
- LC connectivity patterns are associated with cognitive performance and tau pathology in the entorhinal cortex.
- We introduced a novel marker capturing LC connectivity deviations and that is associated with tau accumulation in cognitively normal individuals at high risk for AD.

## 1 BACKGROUND

The locus coeruleus (LC) is a small but essential structure in the brainstem that distributes norepinephrine throughout the brain via its extensive connections.^1^ By modulating neural activity, these connections play a key role in arousal, attention, memory, sleep, emotion, and stress regulation functions.^1^, ^2^, ^3^, ^4^ This neuromodulatory role is crucial for maintaining cognitive functions, allowing the brain to dynamically adjust to changing demands.^5^

Recent animal studies show that the LC is not a uniform structure but consists of distinct groups of neurons that project to different brain regions and serve different functions.^1^, ^6^, ^7^ In rats, it has been shown that the rostral part of the LC mainly projects to frontal cortex, thalamus, and hypothalamus,^8^, ^9^, ^10^ while caudal neurons project to the spinal cord. In macaques, rostral neurons project to thalamus, hypothalamus, cortex, and forebrain, while caudal neurons project into the cerebellum and spinal cord.^3^ In both cases, rostral projections are implicated in the regulation of attention and circadian rhythms, whereas neurons in the caudal LC are involved in respiratory, cardiovascular, and gastrointestinal functions.^3^, ^11^ In humans, the organization of its connections is not well understood. The rostral LC, preferentially affected by ageing and Alzheimer's disease (AD), is primarily associated with memory, mood, and behavior (apathy), and projects to limbic and neocortical regions, whereas the caudal LC, more affected in Parkinson's disease (PD),^12^, ^13^ supports autonomic and general cognitive functions, projecting mainly to the cerebellum and spinal cord.^14^, ^15^ However, this simple subdivision does not capture the functional complexity of the LC.^14^, ^15^ This is particularly important in the context of aging and AD, both of which are known to affect LC function and norepinephrine production.^11^ In AD, the LC is the first brain region to accumulate tau pathology^16^ and plays a key role in regulating memory.^17^ Furthermore, because of its widespread connections, the LC has been proposed as a driver for the spread of tau pathology from the brainstem to the cortex in early AD stages.^1^

Although several studies have identified a loss of signal within the LC both in aging and AD,^18^, ^19^ it is currently unclear which LC changes are specific to each condition. The distinction between normal aging and early disease processes is important for identifying early markers of vulnerability as well as potential resilience that can be incorporated into prevention strategies. To address these questions, we investigated the organization of LC connectivity across both aging and AD using a gradient‐based approach.^20^ This method has previously been employed to investigate the topography of other subcortical nuclei that, like the LC, are challenging to measure due to their small size.^21^ Moreover, we have previously characterized the complex LC subregional functional patterns in aging^22^ and PD.^23^ However, the anatomical organization of the LC has not been previously studied with gradient approaches. Furthermore, these spatial patterns have been shown to provide new insights into the connectivity organization during aging and disease.^21^, ^22^

Our findings revealed a novel organization of LC connections that showed common alterations between aging and the AD continuum or alterations that were specific only to the AD continuum. These changes were linked to a broad range of functions in aging and key clinical measures in AD, reinforcing LC's role as a central neuromodulatory hub. AD‐specific changes were connected to synaptic and presenilin genetic profiles and were strongly associated with tau burden in the entorhinal cortex—an early site of AD pathology^24^ and a key target of LC projections. Interestingly, our proposed new imaging marker (LC gap), was able to predict with high accuracy which cognitively normal individuals were less likely to accumulate tau over time. Together, these results highlight the critical role of LC connections in cognition, both in aging and AD, and suggest that the LC gap may serve as a promising marker of resilience against AD‐related pathology.

**RESEARCH IN CONTEXT**

1. **Systematic review**: We searched PubMed and recent conference abstracts for studies on locus coeruleus (LC) connectivity, aging, and Alzheimer's disease (AD). Previous studies showed that the LC's connections play a role in cognition and deteriorate both in aging and AD. However, these studies mostly focused on specific white‐matter tracts, leaving the whole‐brain LC's connectivity underexplored.
2. **Interpretation**: Our findings identify specific and shared alterations in whole‐brain LC projections across the AD continuum and aging. Given the associations between LC connectivity, cognition, and tau pathology, these findings provide a basis for developing markers of resilience against AD‐related neurodegeneration. Since distinguishing normal aging from early disease processes is essential for identifying early vulnerability and resilience, our study highlights the potential of LC connectivity measures to inform prevention‐focused strategies.
3. **Future directions**: To determine how LC's anatomical connections change over time and track individual disease trajectories, future studies should aim to validate our findings in longitudinal and more heterogeneous cohorts.

## 2 METHODS

### 2.1 Cambridge Centre for Ageing and Neuroscience cohort

A total of 640 cognitively normal individuals between 18 and 88 years old were included from the Cambridge Centre for Ageing and Neuroscience (Cam‐CAN) dataset (https://www.cam‐can.org) who underwent brain imaging, consisting of diffusion‐weighted imaging (DWI) and T1‐weighted (T1W) magnetic resonance imaging (MRI), and an extensive battery of cognitive, motor, and other behavioral tests (see Table 1). Using age‐binned windows, we generated gradient maps for 71 discrete age samples between 18 and 88 years with a window sample size distribution as shown in Figure S1. Participants were recruited from a larger population‐based sample derived from Primary Care Trust lists, ensuring a diverse and representative sample and reporting the family history of some health problems (e.g., heart disease, stroke, and diabetes). Participants were included if they were willing to continue with the study, be cognitively healthy with a Mini‐Mental Status Examination (MMSE) score greater than 24, meet hearing, vision, and English language ability criteria necessary for completing experimental tasks, and be free of MRI or magnetoencephalography (MEG) contraindications and neurological or serious psychiatric conditions.^25^

**TABLE 1:** Summary of individual characteristics and cognitive test performances in Cam‐CAN dataset.

Variables	*n*	Median (interquartile range)
Age (years)	640	55 (32)
Sex (male/female)	(318/322)	–
Education (years)	633	15 (6)
Cattell Culture Fair	615	32 (10)
Famous Faces Recognition	614	25 (8)
Hotel‐task	613	268.16 (254.27)
Benton test of Facial Recognition	612	23 (4)
Picture–Picture Priming	602	328 (48)
Force Matching	293	0.16 (1.32)
Emotional regulation—Reactivity	261	5.57(2.23)
Emotional regulation—Reappraisal	261	0 (1.2)
Ekman's Emotion Hexagon test	616	90 (25)
PSQI	604	3 (4)
HADS Anxiety	629	5 (4)
HADS Depression	629	2 (3)
T1W‐WMH (mm^3^)	627	1079.41 (1714.19)
Cardiovascular risk (Yes/No)	(192/435)	0 (1)

*Note*: The size of the sample (*n*) for each variable (except for the sex and cardiovascular risk) is shown in the first column. The median followed by the interquartile range in parenthesis of the values for each variable (except for the sex) are shown in the second column. All participants underwent a comprehensive battery of assessments covering global cognition (Cattell Culture Fair), memory (Famous Faces Recognition), executive functions (Hotel‐task), visuoperceptual functions (Benton Test of Facial Recognition), language (Picture‐picture priming), motor skills (Force Matching task), emotional regulation (reactivity and reappraisal), emotion expression recognition (Ekman's Emotion Hexagon test), sleep assessed with the Pittsburgh Sleep Quality Index (PSQI), as well as anxiety and depression measured with the Hospital Anxiety and Depression Scale (HADS). Cardiovascular risks and vascular pathology, measured with white matter hyperintensities (WMH) in T1‐weighted imaging (T1W), were also recorded.

All subjects completed a wide battery of tests that assessed global cognition (Cattell Culture Fair), memory (Famous Faces Recognition), executive functions (Hotel‐task), visuoperceptual functions (Benton Test of Facial Recognition), language (Picture‐picture priming), motor skills (Force Matching task), emotional reactivity and reappraisal, emotion expression recognition (Ekman's Emotion Hexagon test), sleep assessed with the Pittsburgh Sleep Quality Index (PSQI), as well as anxiety and depression measured with the Hospital Anxiety and Depression Scale (HADS). Emotional regulation was also evaluated in another stage of the study in a subset of participants.^25^, ^26^ Additionally, data on cardiovascular symptoms such as diabetes, hypertension, hyperlipidemia, high blood pressure, or other cardiac disturbances (e.g., arrhythmia, palpitations, or irregular heartbeat) were collected. For analysis, the presence of any cardiovascular symptom was summarized as a single binary variable indicating whether the individual reported experiencing any of these conditions (yes/no).

Written informed consent was provided by all subjects after the study received ethical approval from the Cambridgeshire 2 Research Ethics Committee (reference: 10/H0308/50) and the adherence to the Helsinki Declaration was verified.

### 2.2 Alzheimer's Disease Neuroimaging Initiative cohort

Data used in the preparation of this article were obtained from the Alzheimer's Disease Neuroimaging Initiative (ADNI) database (adni.loni.usc.edu). The ADNI was launched in 2003 as a public–private partnership, led by Principal Investigator Michael W. Weiner, MD. The primary goal of ADNI has been to test whether serial MRI, positron emission tomography (PET), other biological markers, and clinical and neuropsychological assessment can be combined to measure the progression of mild cognitive impairment (MCI) and early AD. The data were used in the present study to assess differences in LC connectivity along the AD continuum. The ADNI cohort included 132 participants across the AD continuum (cognitively normal [CN], MCI, and AD dementia) that were divided into four groups (see Table 2) based on amyloid‐β pathology (Aβ) and cognitive status: cognitively normal individuals with amyloid‐β negativity (Aβ‐) and individuals with amyloid‐β pathology (Aβ+) at baseline that were CN, with MCI, or with early AD. The final sample consisted of 77 CN Aβ−, 16 CN Aβ+, 31 MCI Aβ+, and 8 AD Aβ+ individuals (Table 2 and Figure S1). Amyloid pathology was established using cerebrospinal fluid (CSF) Aβ1‐42 levels with a cutoff of ≤1100 pg/ml for positive and > 1100 pg/ml for negative cases.^27^

**TABLE 2:** Summary of individual characteristics of ADNI dataset.

Variables	CN Aβ‐ (*n* = 77)	CN Aβ+ (*n* = 16)	MCI Aβ+ (*n* = 31)	AD Aβ+ (*n* = 8)
Age (years)	69.3 (8.4)	70.8 (5.25)	73.6 (11.425)	76.15 (6.5)
Sex (male/female)	32/45	7/9	16/15	5/3
Education (years)	18 (2)	16 (4)	16 (4)	15.5 (3.5)
Tau‐PET SUVR	1.2150 (0.0785)	1.2079 (0.0772)	1.3891 (0.1684)	1.8904 (0.19)
MMSE	29 (2)	29 (2.5)	27 (3)	19 (9.5)
mPACCtrailsB	1.01 (2.92)	−1.05 (7.56)	−7.07 (7.10)	−19.19 (12.18)
ADAS‐Q4	2 (2)	3 (3)	7 (4)	8 (1)
FLAIR‐WMH (mm^3^)	1838.72 (3481.84)	2208.2 (1877.98)	4581.0 (8550.01)	5037 (5621.26)
Cardiovascular risk (yes/no)	55/22	16/0	22/9	6/2

*Note*: The sample size (*n*) is shown in parenthesis for each group. The values in the table represent the medians followed by the interquartile range in parenthesis for each group (except for sex and cardiovascular risk). Variables included age, sex, education, baseline tau pathology measured with the standardized uptake value ratio (SUVR) using positron emission tomography (PET) imaging, Mini‐Mental State Examination (MMSE) score for global cognition, a modified Preclinical Alzheimer's Cognitive Composite that used the Trail Making Test B (mPACCtrailsB) and the Alzheimer's Disease Assessment Scale‐Cognitive (ADAS‐Cog) Cognitive Subscale—four‐item version (ADAS‐Q4) for memory. Cardiovascular risks and Vascular pathology quantified with white matter hyperintensities (WMH) in fluid‐attenuated inversion recovery (FLAIR) imaging were also employed.

For the purposes of this project, participants with similar DWI parameters, T1W MRI, ^18^F‐Flortaucipir (AV‐1451) PET scans and CSF biomarkers were included. Inclusion criteria for CN individuals were MMSE scores from 24 to 30, a Clinical Dementia Rating ‐ Sum of Boxes (CDR‐SB) score of 0, and no signs of depression, MCI, or dementia. MCI participants are classified based on Petersen's criteria, while probable AD cases meet the National Institute of Neurological and Communicative Disorders and Stroke and the Alzheimer's Disease and Related Disorders Association (NINCDS/ADRDA) standards, with MMSE scores ranging from 18 to 26 and CDR‐SB scores between 0.5 and 1.0. Exclusion criteria for all subjects include structural brain abnormalities, significant neurological disorders other than AD, or the use of psychotropic medications affecting memory.

Cognitive function was assessed using MMSE score for global cognition, a modified Preclinical Alzheimer's Cognitive Composite that used the Trail Making Test B (mPACCtrailsB) and the Alzheimer's Disease Assessment Scale‐Cognitive (ADAS‐Cog) Cognitive Subscale, four‐item version (ADAS‐Q4) for memory. Additional data on the presence of cardiovascular symptoms and total volume of white matter hyperintensities (WMH) were also provided.

The ADNI study operates in compliance with institutional research committees, Good Clinical Practice guidelines, the Declaration of Helsinki (1975) and its updates, as well as U.S. regulations on human subjects and Health Insurance Portability and Accountability Act (HIPAA) standards. Written informed consent was obtained from all participants or their representatives, with ethical approval granted at each participating ADNI site. As a multicenter, open‐access initiative with data collected from over 50 sites across the United States and Canada, ADNI aims to identify biomarkers of AD progression. Researchers who agree with ADNI's data use policies have access to de‐identified data strictly for scientific research, teaching, or clinical planning, with clear guidelines prohibiting any re‐identification of participants. ADNI was launched in 2003 as a public–private partnership, led by Principal Investigator Michael W. Weiner, MD. The primary goal of ADNI has been to test whether serial MRI, PET, other biological markers, and clinical and neuropsychological assessment can be combined to measure the progression of MCI and early AD.

### 2.3 Image acquisition

All individuals from Cam‐CAN underwent an imaging protocol that included DWI and T1W sequences. The DWI were acquired using the following parameters: 30 diffusion gradient directions for two different b values (1000 and 2000 s/mm^2^), repetition time (TR) = 9100 ms, echo time (TE) = 104 ms, voxel size = 2 mm isotropic, field of view (FOV) = 192 × 192 mm, 66 axial slices and number of averages = 1. T1W structural imaging was acquired with Magnetization Prepared RApid Gradient Echo (MPRAGE) sequence with TR = 2250 ms; TE = 2.99 ms; inversion time (TI) = 900 ms; flip angle = 9 degrees; FOV = 256 × 240 × 192 mm; voxel size = 1 mm isotropic; generalized autocalibrating partially parallel acquisitions (GRAPPA) acceleration factor = 2; acquisition time of 4 minutes and 32 seconds.

Participants included in the ADNI cohort underwent DWI, T1W, and ^18^F‐Flortaucipir (AV‐1451) PET imaging. DWI were acquired with 48 directions with b = 1000 s/mm^2^, TR = 7200 ms, TE = 56 ms, voxel size = 2 mm isotropic, FOV = 232 mm × 232 mm × 160 mm. Furthermore, T1W sequences were acquired with MPRAGE sequence with TR = 2300 ms; TE = min full echo; TI = 900 ms; flip angle = 9 degrees; FOV = 256 mm × 240 mm × 208 mm; voxel size = 1 mm isotropic and acceleration factor of 2. Finally, tau‐PET ^18^F‐Flortaucipir (AV‐1451) scans were acquired with a 30‐minute dynamic scan consisting of six 5‐minute frames, starting 75 minutes after an injection of roughly 10 mCi dose of [^18^F]‐AV1451 (370 MBq).

### 2.4 Image preprocessing

All DWI scans were preprocessed using the FSL toolbox to identify the white matter connections between the LC and the rest of the brain (v.6.0.5,^28^). First, DWI volumes were brain‐extracted using FSL BET^29^ and corrected for motion and eddy current artifacts with FSL eddy.^30^ Preprocessed scans were aligned to Montreal Neurological Institute (MNI) template space using non‐linear registration with FSL FNIRT.^28^ Fiber orientation was estimated voxel‐wise using a ball‐and‐stick model adjusted for multi‐shell diffusion data as implemented in FSL bedpostX.^31^ The LC structure was delineated using a consensus mask from a meta‐analysis study.^32^ Probabilistic fiber tracking (measured with fractional anisotropy) was performed with FSL probtrackx2^33^ from each LC voxel to 246 cortical and subcortical ROIs in both hemispheres defined by the Brainnetome parcellation.^34^ For each seed voxel, 5000 streamline samples were drawn to approximate the voxel‐specific connectivity distribution. A connectivity vector was built for each seed voxel by counting the number of streamlines reaching each target region. Streamline counts were normalized using the pathway length to account for the overestimation bias towards more proximal connections.^35^

To quantify the presence of vascular pathology, we quantified the volume of WMH. In the Cam‐CAN cohort, T1W images were preprocessed using FastSurfer,^36^ a fast and accurate deep learning pipeline FreeSurfer (http://surfer.nmr.mgh.harvard.edu/) alternative. The pre‐processing steps are the same as standard steps such as motion correction, alignment of brain to standard space, spatial normalization, brain extraction, bias field correction, and subcortical and white matter segmentations. White matter segmentation from the pre‐processed T1W MRI was then used to obtain WMH using the WMH‐SynthSeg tool that offers segmentations for WMH or hypointensities, in T1W imaging.^37^ In the ADNI cohort, WMH are automatically detected from preprocessed fluid‐attenuated inversion recovery (FLAIR) images as documented on the ADNI methods.^38^

Finally, ^18^F‐Flortaucipir (AV‐1451) PET images in the ADNI cohort were preprocessed by applying the following steps:^1^ correction of patient motion by co‐registering each 5‐minute frame to the first acquired raw image file;^2^ average of the 5‐minute frames;^3^ standardization of image and voxel size and^4^ smoothing of images to uniform resolution Then, preprocessed PET images were co‐registered to the T1W sequence to obtain standardized uptake value ratio (SUVR) values (normalized to the cerebellar gray matter) for each brain region, as documented on the ADNI methods (https://adni.loni.usc.edu/data‐samples/adni‐data/neuroimaging/pet/). We used the mean ^18^F‐Flortaucipir SUVR values that were available and normalized to the cerebellar gray matter.

### 2.5 Computation of LC gradients

The topography of LC structural connectivity was estimated using connectopic mapping.^20^ A voxel‐wise similarity matrix of the LC was built for each subject using the structural connectivity vectors derived from tractography results. The eta‐squared similarity measure was used to quantify the similarity between structural connectivity vectors, resulting in a ROI voxel by ROI voxel similarity matrix.^39^ All participants were then grouped, either according to age in the Cam‐CAN dataset (71 1‐year age bins, spanning 1 to 20 subjects per bin) or Aβ and cognitive status (CN Aβ−, CN Aβ+, MCI Aβ+, AD Aβ+) in the ADNI dataset. Because ADNI groups varied considerably in sample size (77 CN Aβ−, 16 CN Aβ+, 31 MCI Aβ+, and eight AD Aβ+ individuals), each group underwent 100 bootstrap iterations where 20 participants were sampled with replacement. The distribution of participants contributing to each age bin or pathology/cognitive status is reported in Figure S1. This window size was chosen to balance two constraints: ensuring sufficient statistical power and stability for estimating gradients while accommodating the limited sample sizes present in some ADNI subgroups.^22^ Finally, each group of similarity matrices (age bins in the Cam‐CAN dataset or bootstrap iteration in the ADNI dataset) was transformed into a multilayer connected graph and decomposed into connectivity eigenmodes with the Laplacian eigenmaps algorithm. The eigenmodes represent axes along which the largest stepwise change in structural connectivity occurs. This way, spatially graded patterns of connectivity organization (gradients) can be obtained, along which voxels with similar anatomical connectivity fingerprints are positioned close to each other. The dominant connectivity gradient extracted from each age bin (Cam‑CAN) or bootstrap iteration (ADNI) was then used for subsequent analyses. The whole‑brain map of the dominant gradient for the CN older subgroup (age 55–86 years) in both the aging and AD continuum cohorts can be seen in Figure S2. To determine the extent to which the LC similarity matrix is captured by the dominant gradient, we estimated the hypersphere radius and corresponding root‐mean‐square deviation (RMSD) across increasing Euclidean embedding dimensionalities.^40^, ^41^ In both cohorts, RMSD decreased sharply from dimension 1 to 2 and plateaued thereafter (Figure S2), indicating that the similarity matrix is effectively low‐dimensional and largely dominated by a single principal axis of variation (dominant gradient). We then quantified the dominant gradient's explained variance by dividing its associated Fiedler eigenvalue by the sum of all non‐zero Laplacian eigenvalues,^42^ showing that it explains 25% of the total variance in ADNI and 24% in Cam‐CAN (CN participants aged 55–86).

To characterize the spatial features of these connectivity gradients, a third‐order trend surface model (TSM) was fitted using a Bayesian linear regression framework based on previous studies.^21^, ^22^ The TSM describes the spatial layout of the gradients using a data‐driven set of spatial basis functions. This results in nine coefficients summarizing a third‐order polynomial defining the spatial variation of the structural connectivity gradients in the X, Y, and Z directions of MNI152 coordinate space. These nine parameters are the Bayesian linear regression coefficients (x, y, z), and their second‐ and third‐power coefficients (*x^2^*, *y^2^*, *z^2^*, and *x^3^*, *y^3^*, *z^3^*). First‐order parameters of the modeled surface describe the changes in a flat plane, while second and third‐order parameters describe non‐linear spatial changes like curvature.^22^

To characterize connectivity profile differences along the gradient, the average normalized gradient was thresholded at 0.5 to obtain masks of dorsal and ventral LC regions. Probabilistic tractography was re‐run for dorsal and ventral LC masks for all participants as described in section Image preprocessing. Resulting projections were categorized as either projecting from either the dorsal part or ventral part based on the mode of maximal correlation.

### 2.6 Statistical analysis

All associations between gradient parameters and other variables were analyzed using a windowed correlation approach to avoid inconsistency of individual gradients. At the individual level, gradients showed variability in the orientation of their dominant gradient direction, possibly due to the small size of the LC and the low resolution, leading to inconsistent and non‑comparable gradient maps. In the Cam‐CAN cohort, gradient maps were computed within age‐binned windows, resulting in a dataset comprising 71 age‐specific samples ranging from 18 to 88 years old with window sample size distributions shown in Figure S1. For the ADNI cohort, gradients were estimated separately for each diagnostic group along the AD continuum consisting of 77 CN Aβ−, 16 CN Aβ+, 31 MCI Aβ+, and eight AD Aβ+ participants (Table 2 and Figure S1). Given the unequal group sizes, a bootstrap resampling procedure with 100 iterations was applied to mitigate sample size imbalances. In each iteration, 20 participants were randomly selected with replacement per group to calculate the subset‐level gradient, along with their associated behavioral and other relevant variables that were resampled and averaged in parallel to generate a representative dataset. For each age bin or bootstrap iteration, age and education were represented by the mean across all included participants. Sex was represented by a variable defined as the proportion of males within each bin or iteration, calculated as the number of males divided by the total number of males and females. To control for differences in scale, range, and property differences between modalities, all variables used in the association analyses were z‐scored prior to analysis.

#### 2.6.1 Associations between LC gradients and age

To test the association between the LC gradients and age in the Cam‐CAN cohort, we computed the Spearman partial correlation between the nine TSM parameters and age while correcting for sex and education. Results were corrected for multiple comparisons using the false discovery rate (FDR) method with a false discovery control level α = 0.01. To identify the point at which the correlation between the TSM parameters and age in Cam‐CAN becomes more pronounced, we applied a rolling correlation analysis with derivative‐based breakpoint detection. A moving window approach (window size = 15) captured local correlation changes, while the first and second derivatives quantified the rate and acceleration of the correlation, respectively. The breakpoint was determined as the point where the second derivative reached its minimum or maximum (depending on the sign of the correlation), marking a significant shift in the correlation pattern. Importantly, this rolling analysis was performed on the 71 age‑bin gradient, each representing a distinct, non‑overlapping group of participants, such that each window of 15 values reflects correlations across aggregated age‑specific samples rather than individual subjects.

#### 2.6.2 Associations between LC gradients and AD

To assess the changes in the LC gradients parameters along the AD continuum within the ADNI cohort, we divided the groups based on amyloid pathology and cognitive status: CN Aβ+, MCI Aβ+, and AD Aβ+. Then, Kruskal‐Wallis tests, followed by post hoc pairwise comparisons (P_FDR _< 0.01), were used to test the differences in the gradient parameters between the different groups.

#### 2.6.3 Associations between LC gradients and cognitive functioning

The relationship between the gradient parameters with cognition was evaluated using partial least squares (PLS) analysis^43^ in the two cohorts. This statistical technique linearly decomposes the predictor and predicted variable matrix into latent variables (LVs), which are optimized to maximize the covariance between the resulting predictor and predicted matrix components (factors and loadings). A separate PLS model was fitted for each cognitive test, including the LC gradient parameters along with age, sex, education, white matter lesions volume, and the presence of cardiovascular risk factors (previously associated with aging and AD^44^) as predictors. Cross‐validation was used to determine the optimal number of latent variables. We used the variable importance in the projection (VIP) statistic to identify significant predictors, a common measure for estimating variable importance in standard PLS^45^, and a permutation test on the VIP score.^46^ Predictors were considered significant if their VIP score was greater than 1.

#### 2.6.4 Associations between LC gradients and Tau pathology

The association between the LC gradient parameters (*x*, *y*, *z*, *x^2^*, *y^2^*, *z^2^*, *x^3^*, *y^3^*, *z^3^*) and entorhinal tau was calculated using Spearman partial correlations while correcting for age, sex, and education (P_FDR _< 0.01) in the ADNI cohort. Furthermore, linear regression models were employed to compare the predictive power of LC gradients and entorhinal tau in estimating cognitive performance (mPACCtrailsB) as well as to assess the predictive efficacy of the LC gradients and entorhinal tau in predicting each other. All models incorporated age, sex, and education as covariates and were evaluated 10,000 times with random cross validation partition where 80% of the dataset was used to build the model and 20% was used to test the model.

### 2.7 Transcriptomic analysis

Brain‐wide gene expression atlases, which assess the transcriptional activity of thousands of genes in various anatomical sites, have recently been constructed. In earlier studies, the distributed features of connectome structure and function were linked to regional distributed features in gene expression.^47^ Here, we used the Allen Human Brain Atlas (AHBA), a brain‐wide database of gene transcription activity, to determine if the variations in rostral LC connectivity between aging and AD were linked to certain gene expression profiles. Specifically, the dataset included transcriptomic data for 15,633 genes derived from 1285 cortical samples from six *post‐mortem* donors that were mapped onto the Brainnetome atlas based on their MNI coordinates.^48^, ^49^

The connectivity differences between the Cam‐CAN and ADNI cohorts were then calculated using a map we generated of the specific LC voxel‐wise projections affected in AD. In order to correlate these connectivity differences with gene expression patterns and rank genes according to their biological significance, we used PLS regression.^50^ The PLS analyses identifies the association between the weighted linear combinations (i.e., components) of transcription patterns across these 15,633 genes with the between‐group differences in LC connectivity. To determine the contribution of the different genes to the relationship with the altered LC connectivity, we ranked the list of genes according to their first PLS component (PLS1) weight values. Then, we performed gene ontology (GO) enrichment analysis on the top 1,000 ranked genes to identify enriched categories, applying (P_FDR _< 0.01) with ClusterProfiler.^51^ The GO enrichment results were visualized using the DOSE R library's dotplot and cnetplot (Gene‐Concept Network) functions. Furthermore, we conducted an Over‐Representation Analysis (ORA) using functions from the PANTHER 19.0,^52^ leveraging its predefined pathway annotations to assess whether genes within each module were significantly enriched for known pathways or functions (P_FDR_ < 0.05).

### 2.8 Calculation of LC gap

To compute the LC gap in the AD continuum dataset, based on the previous concept of “brain–age gap”,^53^ we used nine independent linear regression models to fit each TSM gradient parameter (*x*, *y*, *z*, *x^2^*, *y^2^*, *z^2^*, *x^3^*, *y^3^*, *z^3^*) using age, sex, and education as predictors. The difference between the predicted and the individual's actual gradient parameter was defined as the LC gap. To account for the bias effect toward the response, where prediction and response are highly correlated, we applied a correction previously used in brain–age gap studies.^54^ The correction is applied to the predictions by first modeling the relationship between the predicted gradient (Y) and ground truth gradient (Ω) using the following equation:

(1) Y=αΩ+β,

where α and β represent the slope and intercept, respectively. The estimated α and β values are then used to adjust the predicted gradient using the formula:

(2) $$\text{Corrected Predicted Gradient}=\text{Predicted Gradient}+\frac{1}{2}\Omega \left(\alpha \Omega +\beta \right).$$

From this, the corrected gradient gap can be computed as:

(3) $$\begin{array}{ccc} \mathrm{Corrected}\;\mathrm{Gradient}\;\mathrm{Gap} & = & \mathrm{Corrected}\;\mathrm{Predicted}\;\mathrm{Gradient}\\ & & -\;\mathrm{Ground}\;\mathrm{Truth}\;\mathrm{Gradient}. \end{array}$$

After applying this correction approach, Spearman partial correlation was used to explore the relationship between the LC gap and longitudinal Tau‐PET SUVR in the entorhinal. Tau pathology accumulation was estimated for each participant by computing the difference in SUVR values between the final and baseline timepoints, divided by the number of days between these assessments to account for individual variability in follow‐up intervals. An additional correlation analysis was conducted to account for potential confounding effects of pre‐existing tau pathology, with the correlation recalculated while adjusting for baseline tau accumulation.

## 3 RESULTS

### 3.1 Participants

This study included two independent cohorts, one from the Cam‐CAN (https://www.cam‐can.org) and another from the ADNI (http://adni.loni.usc.edu). From Cam‐CAN, 640 cognitively normal individuals between 18 and 88 years old were included, whereas 132 subjects across different stages of the AD continuum (cognitively normal, MCI, AD dementia) with (+) or without (‐) Aβ pathology (CN Aβ‐, CN Aβ+, MCI Aβ +, AD Aβ+) were included from ADNI. All participants underwent DWI to identify the anatomical connections projecting from the LC to other cortical and subcortical brain areas of the Brainnetome atlas (Figure S3). These projections are asymmetric with stronger left‐sided connectivity, aligning with previous studies of left‐lateralized LC structure and function that found increased signal intensity and functional connectivity changes in the left LC associated with aging,^12^, ^22^ PD^55^, ^56^ and AD.^57^ Furthermore, subjects from the ADNI cohort underwent longitudinal 18F‐Flortaucipir (AV‐1451) PET over 3.41 ± 1.53 years to measure the accumulation of tau pathology in the entorhinal cortex. For Cam‐CAN, a comprehensive battery of tests assessing global cognition, executive functions, memory, visuoperceptual abilities, language, sensorimotor, and psychiatric functions was included (see the Methods section).^11^, ^58^, ^59^ For ADNI, we included tests commonly used in both research and AD clinical trials assessing global cognition, memory, and a preclinical AD cognitive composite score (see the Methods section).^60^, ^61^

### 3.2 Anatomical LC connections follow a dorsal–ventral connectivity gradient

**FIGURE 1:**
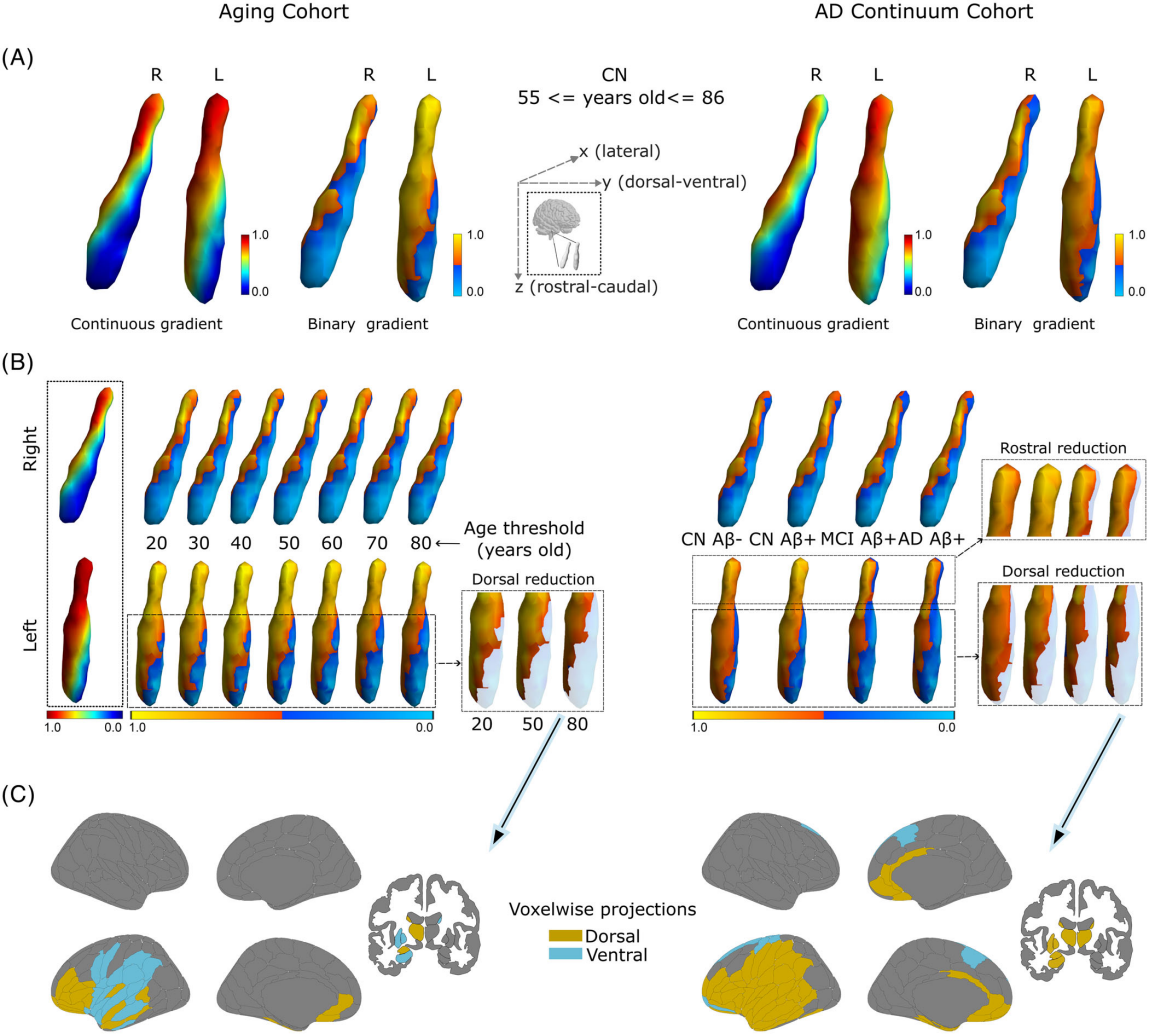
Anatomical gradients of the right (R) and left (L) LC in the aging cohort (Cam‐CAN) and AD continuum cohort (ADNI). (A) Averaged continuous and binary anatomical gradients of LC in CN old group (age range: 55–86 years old) in both aging (left) and AD continuum (right) cohorts. Gradients exhibit same patterns between the two cohorts with a strong correlation (*R* = 0.82, IQR = 0.021). (B) Left: age‐related changes of the LC anatomical gradients in aging dataset. Displayed ages (20, 30, 40, 50, …) represent age thresholds used to average age‑bin gradients for visualization. (e.g., <  20, 20‐30, 30–40, etc.) producing a representative gradient map for each age interval. A loss of the dorsal gradient group of the left LC is observed. Right: changes in anatomical gradients of LC in AD continuum cohort for the four different groups: individuals with Amyloid‐β negativity (Aβ‐) and with Amyloid‐β pathology (Aβ+) at baseline that were cognitively normal (CN), with mild cognitive impairment (MCI) or with early AD. A loss in the rostro‐dorsal part of the left LC is observed. (C) Voxel‐wise projections from affected voxels to the rest of the brain for both the aging (left) and AD (right) cohorts. Regions overlap between the two cohorts and are more numerous in the AD continuum cohort. AD, Alzheimer's disease; ADNI, Alzheimer's Disease Neuroimaging Initiative; Cam‐CAN, Cambridge Centre for Ageing and Neuroscience; CN, cognitively normal; IQR, interquartile range; LC, locus coeruleus.

To identify the anatomical connections of the LC, we applied probabilistic tractography to DWI scans and assessed the organization of these connections with gradient‐based analyses.^20^ First, we built a connectivity matrix for each subject representing the projections from each LC voxel to all other regions in the brain. Then, by comparing the resulting connectomes between the LC voxels, we derived a similarity matrix, which was transformed into a connected graph and decomposed into connectivity eigenmodes using the Laplacian eigenmaps algorithm,^62^ from which the main connectivity gradient of the LC was identified. See the Methods ‐ Computation of LC gradients section for more details. Given that our study was the first to assess the anatomical connectivity gradients of the LC, we measured and visually inspected these gradients in two age‐matched groups of cognitively older adults from Cam‐CAN and ADNI. These groups included 93 cognitively healthy participants from ADNI and 321 participants from Cam‐CAN, all aged 55–86 years. The representative gradients for both groups are shown in Figure 1A, the corresponding DTI projection maps are shown in Figure S3. In Cam‐CAN, we observed that the dominant LC gradient followed an asymmetric dorsal–ventral organization, with stronger connections on the left side. The LC gradient in ADNI was highly consistent with the one observed in Cam‐CAN, showing a strong correlation with the Cam‐CAN gradient (*R* = 0.82, interequartile range [IQR] = 0.021). To further evaluate the reproducibility of this correspondence, we performed a bootstrapping procedure in which we randomly selected 20 participants from each group (with/without replacement) and recomputed the LC gradients for each iteration (see the Methods ‐ Computation of LC gradients section). This procedure was repeated 100 times, and the correlation between gradients obtained from both cohorts was calculated for each iteration. We obtained a high mean correlation between the two gradients (0.87 ± 0.0057), Figure S4), further supporting the robustness and cross‐cohort consistency of the LC gradients. Both gradients proved to be better predictors of aging and cognitive decline than simple connectivity measures, indicating that they provided additional information beyond traditional connectivity methods (Supplementary Analyses).

### 3.3 LC gradients change both across aging and across the AD continuum

To investigate whether the LC gradients change with aging, we divided the participants from the Cam‐CAN cohort into different decade‐based age groups. The visual assessment of these gradients revealed a progressive reduction in connections projecting from the left dorsal LC across the age groups (Figure 1B). A similar pattern was observed across the AD continuum from the ADNI cohort, where we found a progressive loss of connections projecting from the left dorsal LC, with the greatest loss in the AD Aβ+, followed by MCI Aβ+, and then CN Aβ+, when compared to the CN Aβ‐ group (Figure 1B). Moreover, in addition to dorsal LC changes, individuals in the MCI Aβ+ and AD Aβ+ groups also exhibited a loss of connections projecting from the left rostral LC (Figure 1B).

To quantify these spatial changes, we fitted a third‐order TSM^63^ that described changes in the spatial organization of the gradients with 9 coefficients across left‐right (*x*), dorsal–ventral (*y*), and rostral–caudal (z) directions, which were either linear (*x*, *y*, *z*), quadratic (*x^2^*, *y^2^*, *z^2^*), or cubic (*x^3^*, *y^3^*, *z^3^*). TSM parameters condensate high‐dimensional connectopic maps into a small set of spatial coefficients. Lower‐order terms capture linear spatial trends, while higher‐order terms capture non‐linear spatial variations in connectivity organization.^64^ In the aging cohort, significant correlations between age and the TSM gradient's coefficients were found (Table S1). Using a rolling correlation analysis with derivative‐based breakpoint detection, we identified a key transition point around age 49 (Figure S5), suggesting that the LC gradients remain relatively stable through early adulthood but begin to decline in midlife. Moreover, analyses in the AD cohort gradients revealed that the AD Aβ+ group differed significantly from MCI Aβ+ and CN Aβ+ in all gradient directions, whereas MCI Aβ+ could be best distinguished from CN Aβ+ by the dorsal–ventral and rostral–caudal gradient directions (*p* < 0.001, Table S2).

To identify the cortical regions connected to LC subregions affected in aging and AD, we performed voxel‐wise projection analyses between the vulnerable LC voxels and the rest of the brain. Specifically, we examined the dorsal LC region, altered during both aging and AD spectrum, and the rostral LC region, affected only during the different stages of AD. In both cohorts, the LC subregions were anatomically connected to left‐lateralized areas, including the anterior cingulate, lateral frontal, inferior parietal, and insular cortices (Figure 1C). In AD, however, the pattern of altered connectivity was more widespread, extending to additional left frontal, temporal, and sensorimotor regions, as well as the right anterior cingulate and medial frontal cortex. Notably, we observed disrupted connectivity to the left entorhinal cortex—one of the earliest cortical regions affected by tau pathology^24^—only in AD.

Together, these findings show that LC gradients progressively decline across aging and AD, with overlapping changes in dorsal LC connectivity and AD‐specific alterations in rostral connectivity that project to the entorhinal cortex. These changes offer a potential means to differentiate normal aging from early neurodegeneration in the LC.

### 3.4 Changes in LC connectivity in AD are associated with specific transcriptomic profiles

Because AD is influenced by genetic risk factors,^65^ we investigated whether the specific reductions in LC connectivity seen in AD were linked to particular gene expression patterns. To do this, we used gene expression data from the Allen Human Brain Atlas,^48^ mapped onto the Brainnetome atlas, which resulted in a matrix of 15,633 genes across 246 brain regions.

**FIGURE 2:**
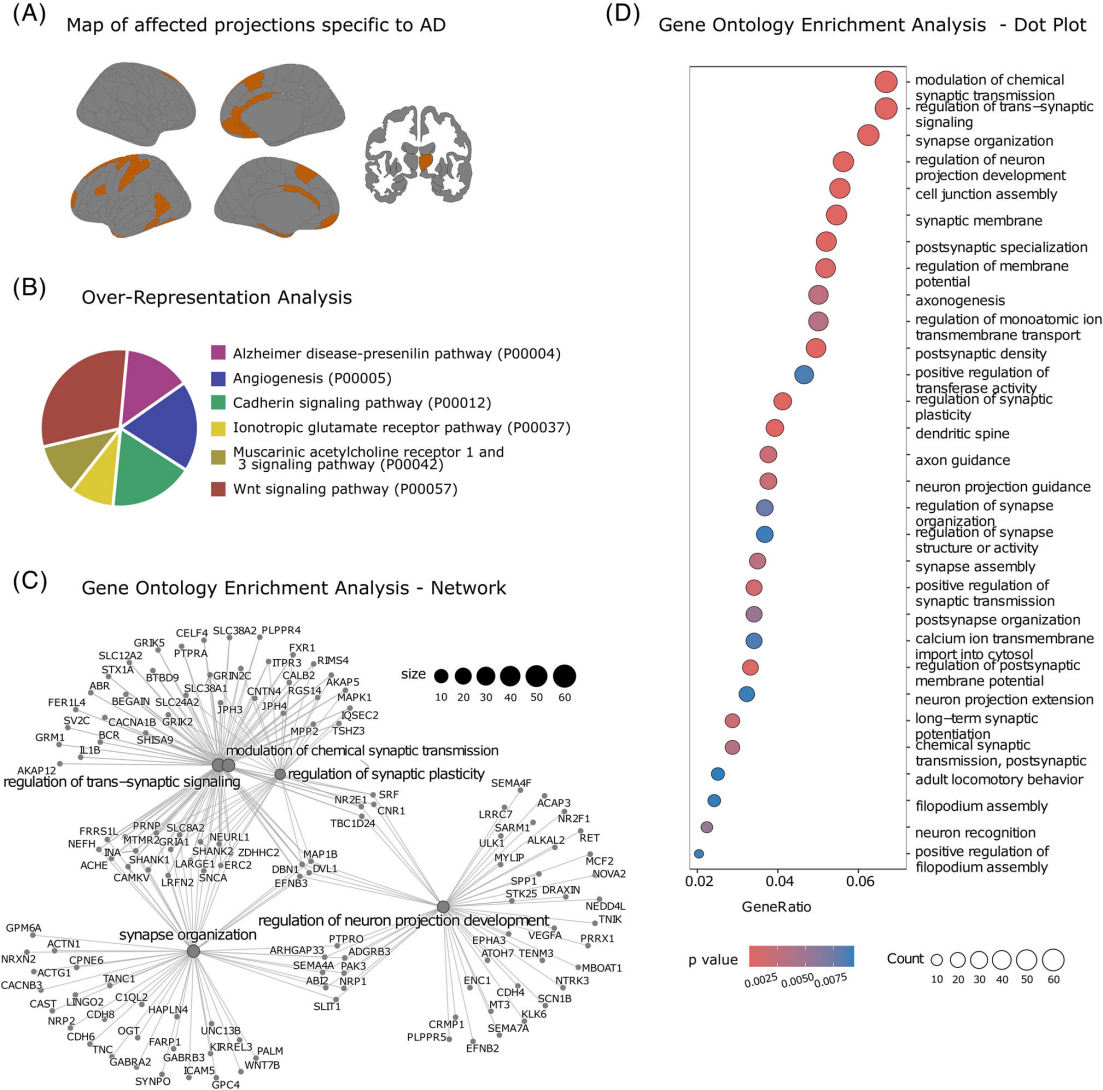
Transcriptomic analysis results. (A) Map of affected voxel‐wise projections specific to AD. The map was used to compute voxel‐wise projection differences within these specific regions between the aging and AD cohorts. (B) Over‐representation analysis (ORA) results showing that the ranking gene set was strongly enriched in pathways related to AD, neurotransmission, and cellular signaling processes (P_FDR _< 0.05). (C) GO enrichment analysis results represented with a modular co‐expression network. Results showed alterations in protein modules associated with synaptic transmission, trans‐synaptic signaling control, neuron projection development, and synapse organization. (D) GO enrichment analysis results represented in a dot plot where several significant terms were identified. These terms were related to neural processes and synaptic function, including modulation of chemical synaptic transmission (GO:005080, *p* < 0.001), regulation of trans‐synaptic signaling (GO:0099177, *p* < 0.001), synaptic organization (GO:0050808, *p* < 0.001), and plasticity (GO:0048167, *p* < 0.001). Additionally, terms related to neuron projection development (GO:0010975, *p* < 0.001), guidance (GO:0097485, *p* < 0.001extension (GO:1990138, *p* < 0.001), and membrane structure (GO:0032589, *p* < 0.001) were also identified. AD, Alzheimer's disease; GO, gene ontology.

We generated a map of the specific LC voxel‐wise projections affected in AD that was then used to compute the projection differences associated with aging (Cam‑CAN) and with AD pathology (ADNI). See the Methods ‐ Transcriptomic Analysis sectionfor details. The transcriptomic analysis was then performed on the difference between these two maps, ensuring that associations were computed exclusively within projections selectively affected in AD. To explore how these differences relate to gene expression, we performed a PLS regression, which linked spatial patterns of gene transcription to the observed LC connectivity differences (Figure 2A).

The PLS1 explained 43.43% of the spatial variance (Figure S6B,C). We tested the spatial correlation between PLS1 and the observed reduction in rostral LC connectivity using a spatial autocorrelation‐corrected permutation test,^66^ finding a significant correlation (*R* = 0.65, *p* < 0.001, *Z* = 2.66, Figure S6D).

To assess the biological relevance of PLS1, we conducted GO and co‐expression network analyses on the top 1,000 genes contributing most to this component. These analyses revealed enrichment in gene networks and GO terms related to neuron projection and synaptic function, including regulation of neuron projection development (GO:0010975), neuron projection guidance (GO:0097485), neuron projection extension (GO:1990138), synaptic organization (GO:0050808), modulation of chemical synaptic transmission (GO:005080), regulation of trans‐synaptic signaling (GO:0099177), and regulation of synaptic plasticity (GO:0048167) (Fold Enrichment > 2.4, *p* < 0.001; Figure 2D–E). Additionally, we found that the AD–presenilin pathway (PANTHER P00004) was significantly overrepresented among these genes (Fold Enrichment = 2.51, *p* < 0.001; Figure 2F).

Together, these results indicate that differences in LC connectivity between aging and AD are strongly associated with gene expression patterns linked to synaptic function, neuron projection morphogenesis and AD‐related pathways.

### 3.5 LC gradients are associated with cognition and behavior in aging and AD continuum

**FIGURE 3:**
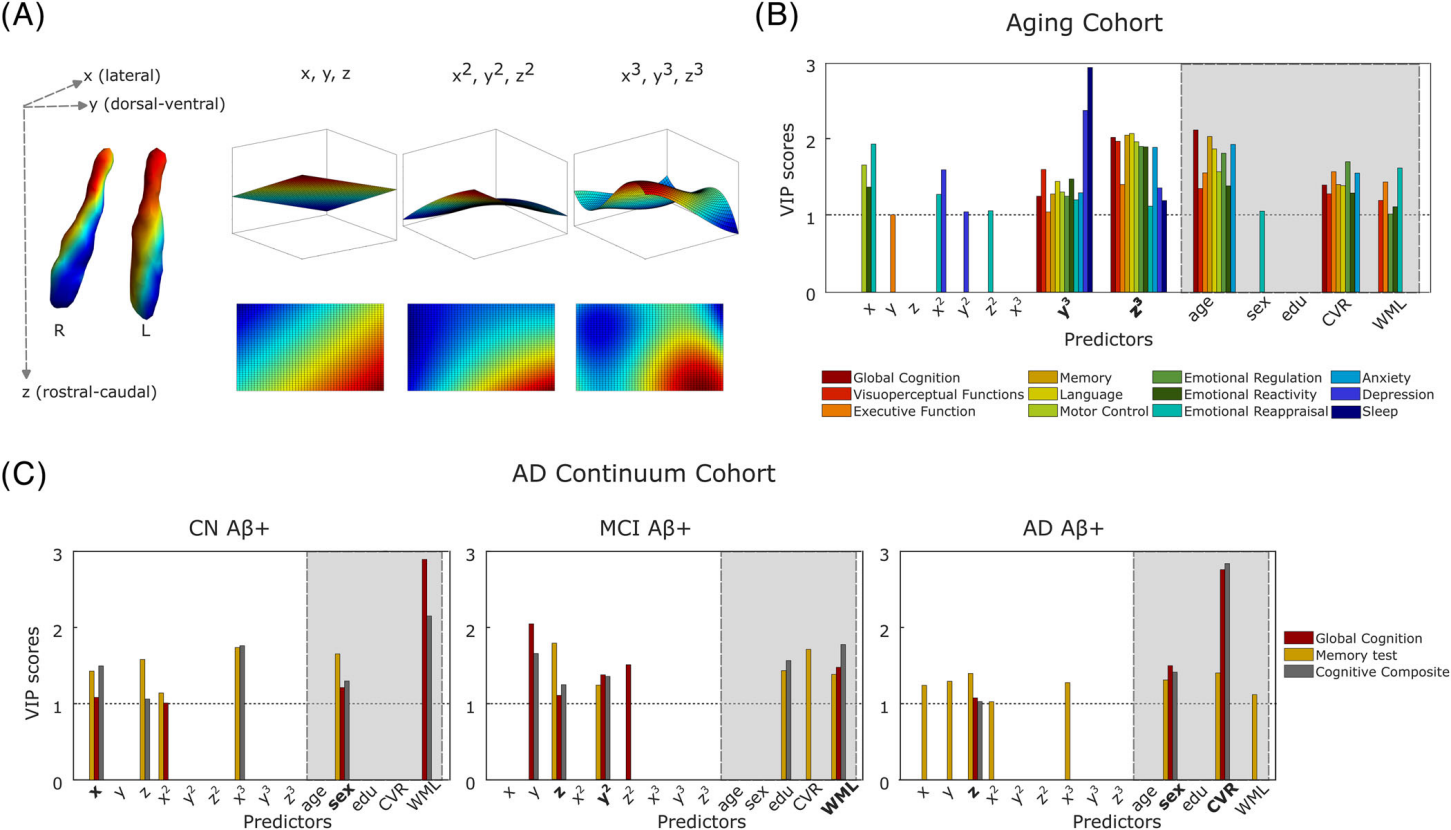
Results from the association between LC gradients with cognition and behavior in aging and across the AD continuum. (A) Representation of trend surface model (TSM) parameters describing the spatial features of the gradient. First‐order TSM coefficients characterize the anatomical connectivity alterations along the right‐left, dorsal–ventral and rostral–caudal directions; first‐order coefficients represent linear changes while higher‐order coefficients represent more complex spatial properties (such as curvature). (B) LC gradients are associated with behavior and cognition in the aging where significant regions are selected based on VIP scores (> 1), highlighting in bold that y^3^ and z^3^ are the important predictors of all tests. (C) Significant predictors of the PLS models for the different cognitive domains are selected based on VIP scores (> 1) for the different groups in the AD continuum cohort: individuals with Amyloid‐β pathology (Aβ+) at baseline that were CN, with MCI or with early AD. Gradient parameters x, z and y^2^ were found as key predictors. In the aging cohort, cubic parameters (y^3^ and z^3^), reflecting more complex changes, emerged as key predictors, whereas in the AD cohort, linear and quadratic parameters (x, z, and y^2^), indicative of simpler changes, were identified as important predictors across all cognitive domains. Age, sex, education, cardiovascular risks, and white matter lesions were included as predictors together with the gradient parameters to control for their effect (displayed within a gray box). AD, Alzheimer's disease; CN, cognitively normal; LC, locus coeruleus; MCI, mild cognitive impairment; VIP, variable importance in the projection.

To understand the relationship between LC gradients and different neuropsychological tests, we conducted a PLS regression analysis using these tests, the nine LC gradient parameters (*x*, *y*, *z*, *x^2^*, *y^2^*, *z^2^*, *x^3^*, *y^3^*, *z^3^*) (Figure 3A) as well as age, sex, education, cardiovascular risk factors, and white matter lesions (WML). The importance of each predictor was assessed using the VIP statistic, a standard measure for estimating variable relevance in PLS models.^45^ Those predictors with VIP > 1 were considered significantly relevant for the model. Our results showed that two gradient parameters, *y^3^* and *z^3^*, were key predictors across all tested domains in the Cam‐CAN aging cohort (Figure 3B). These parameters, which describe complex cubic changes in curvature along the rostro‐caudal and ventral‐dorsal axes, were significantly associated (VIP > 1) with global cognition, executive functions, memory, visuoperceptual skills, language, motor functions, sleep and psychiatric measures, including facial emotion recognition, emotional reactivity, emotion reappraisal, anxiety, and depression.

Interestingly, when similar analyses were performed using cognitive tests across all Aβ+ individuals from the ADNI AD cohort, different gradient parameters emerged as key predictors (Figure 3C). Parameters reflecting simple linear and quadratic changes emerged as significant predictors of cognitive performance (VIP > 1). These parameters were associated with global cognition (CN Aβ+: *x*, *x^2^*; MCI Aβ+: *y*, *y*, *y^2^*, *z^2^*; AD Aβ+: *z*), a preclinical AD cognitive composite score (CN Aβ+: *x*, *z*, *x^3^*; MCI Aβ+: *y*, *z*, *y^2^*; AD Aβ+: *z*), and memory (CN Aβ+: *x*, *z*, *x^2^*, *x^3^*; MCI Aβ+: *z*, *y^2^*; AD Aβ+: *x*, *y*, *z*, *x^2^*, *x^3^*). Specifically, *x*, *z*, and *y^2^*, which describe simple linear and quadratic changes, were consistently identified as important predictors across all cognitive tests.

Together, these findings indicate that LC gradients in both aging and AD are associated with cognitive function while controlling for age, sex, education, cardiovascular risks, and an imaging measure of vascular pathology. However, the specific gradient parameters that serve as key predictors differ between the two. In aging, cognitive performance is primarily linked to higher‐order cubic parameters *y^3^* and *z^3^*, suggesting a more complex, nonlinear pattern of change. In contrast, in AD, cognitive function is best described by simpler, linear and quadratic parameters such as *x*, *z*, and *y^2^* reflecting a different pattern of neurodegeneration progression.

### 3.6 LC gradients are associated with entorhinal tau pathology in AD

To determine whether changes in LC gradients are linked to tau pathology across different stages of AD, we used Spearman partial correlations between tau PET burden in the entorhinal cortex and LC gradient parameters, while correcting for age, sex, and education (P_FDR _< 0.01). Our analysis revealed significant associations between tau pathology and several LC gradient parameters in CN Aβ+ individuals, specifically *x*, *y*, *z*, *x^2^*, *z^2^*, and *z^3^*. However, no significant associations were found in MCI Aβ+ individuals, and in AD Aβ+, only the *z^2^* parameter was significantly correlated with tau burden (Table S3).

**FIGURE 4:**
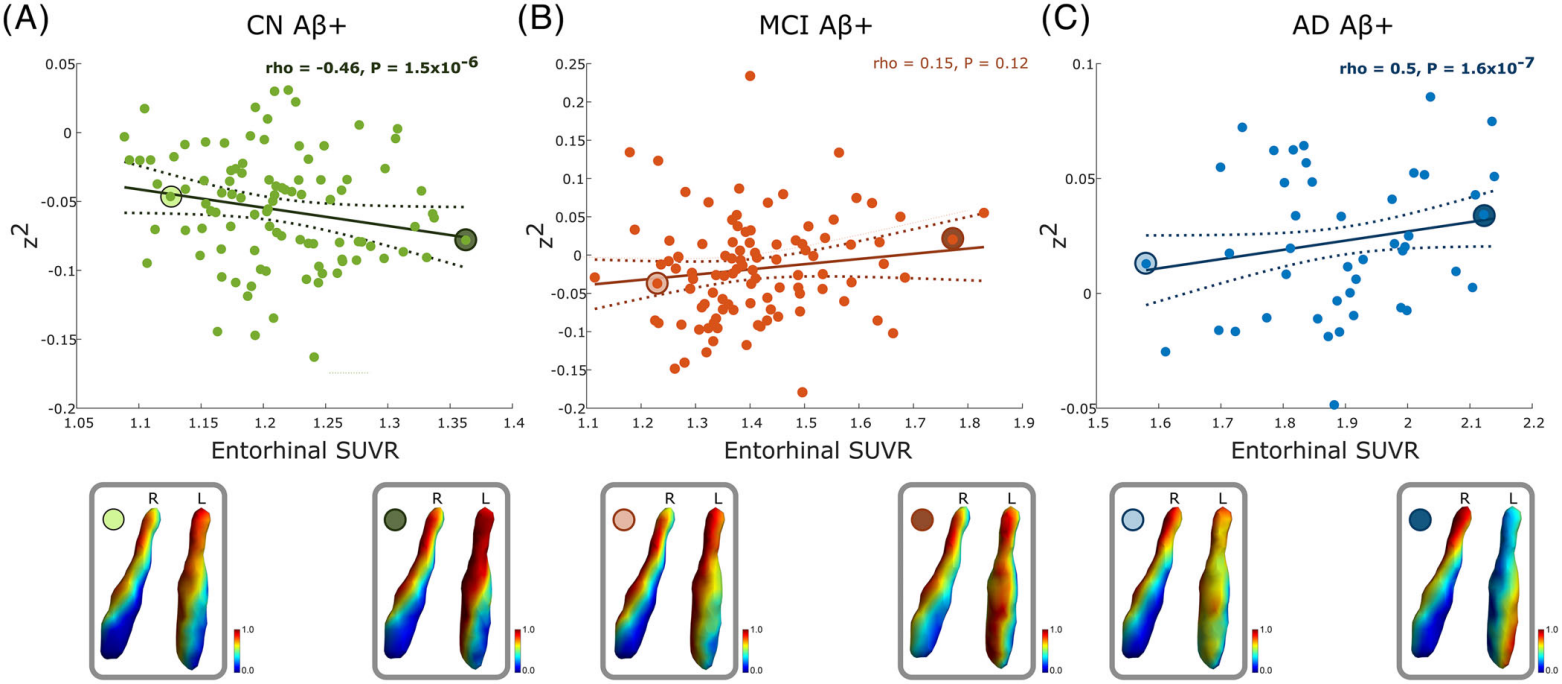
Correlation patterns between entorhinal tau pathology and gradient parameter z2 across the AD continuum. Different individual groups in the ADNI cohort with Amyloid‐β pathology (Aβ+) at baseline that were CN, with MCI or with early AD, were analyzed. (A) Significant negative associations between tau burden in the entorhinal cortex and LC gradient parameter z2 in CN Aβ+ individuals (rho = ‐0.46, *p* < 0.001). This negative correlation reflects a change in dorsal LC connection from weaker to greater as shown in the visualization of the gradients. (B) No significant correlation was found for MCI Aβ+ participants. (C) Significant positive associations between tau burden in the entorhinal cortex and LC gradient parameter z2 in AD Aβ+ individuals (rho = 0.5, *p* < 0.001). This positive correlation was caused by a shift from a pattern where the right LC had a weakened but still relatively intact gradient to a completely reversed gradient. Visualizations of LC gradients in CN Aβ+ and AD Aβ+ individuals with varying tau levels illustrate these opposite correlation patterns. AD, Alzheimer's disease; ADNI, Alzheimer's Disease Neuroimaging Initiative; CN, cognitively normal; LC, locus coeruleus; MCI, mild cognitive impairment.

Further examining *z^2^*, the only LC parameter associated with entorhinal tau levels in both CN Aβ+ and AD Aβ+ groups, revealed opposite correlation patterns between these two groups. In CN Aβ+, *z^2^* was negatively correlated with entorhinal tau levels (rho = ‐0.46, *p* < 0.001), whereas in AD Aβ+, the correlation was positive (rho = 0.5, *p* < 0.001), as illustrated in Figure 4. To better understand these results, we visualized LC gradients in two CN Aβ+ and two AD Aβ+ individuals, each with low and high entorhinal tau levels. In CN Aβ+, the negative correlation between *z^2^* and tau reflected a shift from weaker to stronger dorsal LC connectivity, with increasing levels of tau deposition. In contrast, in AD Aβ+, the positive correlation was driven by a transition from a weakened but relatively preserved gradient, similar to CN Aβ‐, to a pattern where the right LC exhibited a completely reversed gradient, with an increase of tau levels. These findings align with previous evidence suggesting nonlinear connectivity changes across the AD continuum, with a probable compensatory effect in individuals with normal cognition but increasing tau burden.^67^

To test the directionality of the association between LC gradients and tau burden in CN Aβ+, we performed an additional regression analysis (correcting for age, sex, and education). Our results showed that LC gradients were a better predictor of tau pathology in the entorhinal cortex than the entorhinal tau as a predictor of the gradients (Figure S7B), in line with evidence suggesting the LC is the first structure affected in early AD.^68^

### 3.7 Individuals with lower LC gap accumulate tau pathology at a slower rate

**FIGURE 5:**
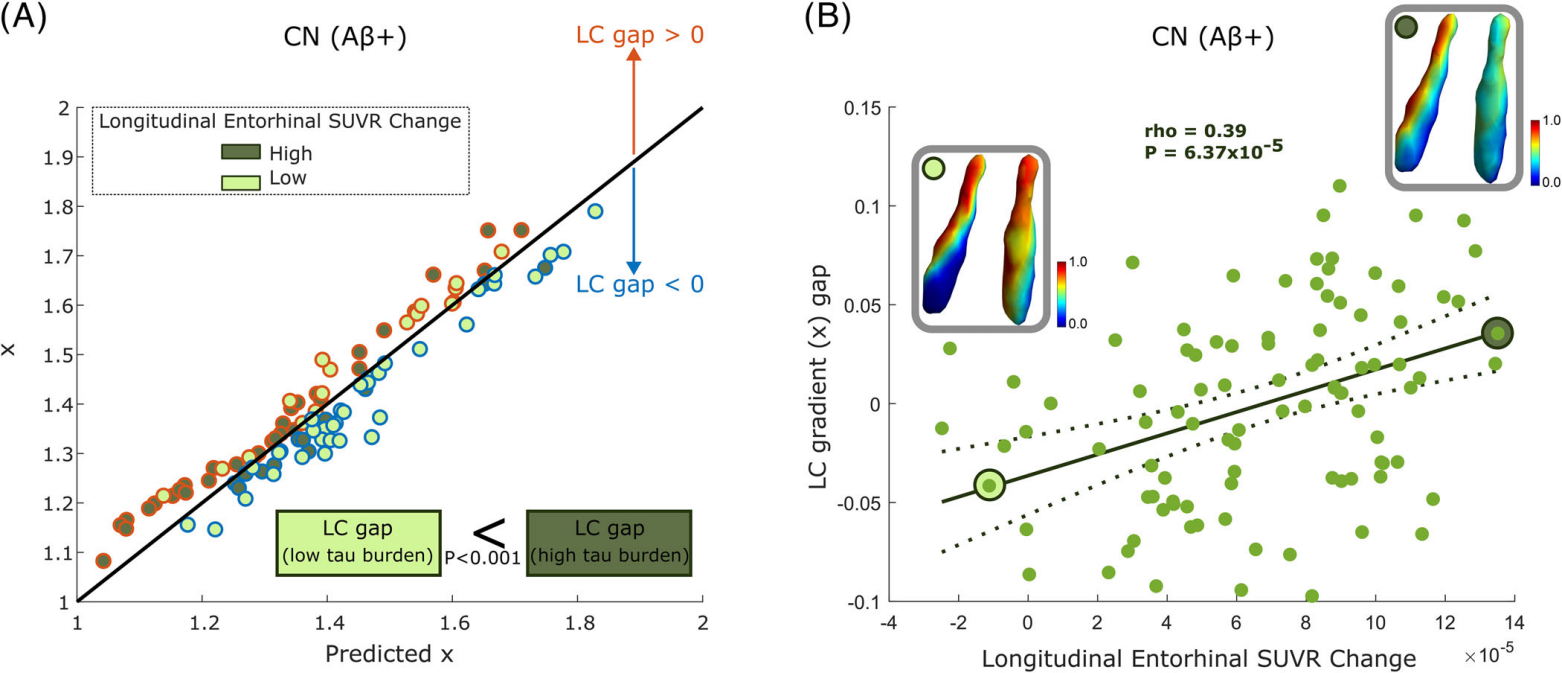
Results of LC gap calculated on parameter x for Amyloid‐β pathology (Aβ+) and CN individuals using linear regression models. (A) The LC gradient gap is significantly positively correlated with longitudinal tau‐PET burden in the entorhinal (rho = 0.39, *p* < 0.001), where a more preserved LC connectivity in the left LC (with less asymmetry between right and left LC) corresponds to a negative LC gap and is associated with a better resilience to early tau pathology accumulation. (B) The scatter plot represents the relation between the gradient parameter x and predicted x. Participants with a low gap (shown in blue) exhibited significantly less longitudinal tau‐PET accumulation in the entorhinal cortex (*p* < 0.001) compared to those with a high gap (shown in red). (C) Changes in LC gradient along the different values of parameter x. An increase in *x* value is associated with strengthened connectivity in left LC and a reduced asymmetry in connectivity between right and left sides of the LC. CN, cognitively normal; LC, locus coeruleus; PET, positron emission tomography.

Similarly, to studies assessing the brain age gap with predictive models,^53^ we calculated the LC gap by applying linear regression models to each LC gradient parameter (*x*, *y*, *z*, *x^2^*, *y^2^*, *z^2^*, *x^3^*, *y^3^*, *z^3^*) and tested their association with entorhinal tau PET in CN Aβ+, MCI Aβ+, and AD Aβ+ groups, separately. The LC gap was defined as the difference between the predicted and the actual gradient parameter, following bias correction between response and prediction.^54^, ^69^ See the Methods ‐ Calculation of LC gap section for more details. Each of these gaps for the nine gradient parameters was then correlated with longitudinal tau accumulation in the entorhinal cortex, while adjusting for age, sex, and education. Only the gap in parameter *x* for CN Aβ+ subjects showed a significant correlation (rho = 0.39, *p* ≤ 0.001) with longitudinal tau‐PET burden in the entorhinal cortex (P_FDR _< 0.01) (Figure 5A). This association remained significant even after adjusting for baseline tau accumulation (rho = 0.41, *p* < 0.001), suggesting that the observed relationship was not solely driven by pre‐existing tau pathology. Compared to participants with high LC gap, participants with a low gap had significantly less longitudinal tau‐PET accumulation in the entorhinal cortex (*p* < 0.001), as shown in Figure 5B. Interestingly, this parameter corresponds to the right‐left direction, potentially reflecting asymmetry between the left and right LC (Figure 5C). In this context, an increased asymmetry—due to affected left LC connectivity—is linked to a larger LC gap and higher early tau pathology accumulation. Finally, this parameter was also associated with cognitive decline in CN Aβ+ individuals (Figure 3C), indicating that it could be a potential resilience marker to early tau pathology in AD.

## 4 DISCUSSION

Through its widespread network of connections, the LC plays a crucial role in coordinating communication between brain regions and shaping behavior. However, the organization of these connections, although previously studied in animals,^1^, ^6^, ^70^ has been overlooked in humans. Here, we found that LC connections follow a reproducible dorsal–ventral organization, with stronger projections from the left side. This organization showed meaningful alterations in aging and AD and was linked to cognitive performance. We also found that a specific pattern of preserved dorsal LC connections was associated with slower tau accumulation over time in individuals at high AD risk. These findings offer new insights into the role of LC in aging and neurodegeneration, suggesting that individual differences may influence resilience to AD‐related pathology.

Although the LC has extensive projections across the brain, most previous studies focused on individual tracts (e.g., ascending noradrenergic or central tegmental pathways).^71^, ^72^ Only one study mapped the whole‐brain LC connectivity, but it was limited to a small sample of 23 young individuals.^73^ In the present study, we aimed to address the gap in how LC connections change across aging and neurodegeneration. We identified a consistent dorsal–ventral gradient of connectivity, providing a more complete view of LC organization that extends beyond traditional rostral–caudal divisions based on neuromelanin‐sensitive imaging.^18^, ^19^ Our findings align with animal studies showing differences in cell morphology, peptide expression, and opposing behavioural effects in the dorsal and ventral LC,^74^ suggesting that LC connectivity gradients could reflect underlying cellular organization.

We observed that the dorsal LC showed connectivity reductions across aging and AD, while rostral LC disruption was specific to the AD stages. This distinction between normal aging or early sign of disease is clinically important, as interventions aim to target individuals in preclinical or at‐risk stages of disease.^75^, ^76^ In addition to the dorsal–ventral organization, we identified a marked asymmetry, with stronger connections from the left LC. This lateralization is consistent with prior imaging studies reporting structural and functional asymmetries of the LC,^12^, ^22^, ^55^, ^77^ as well as higher signal contrast in the left LC.^18^, ^22^ Lateralized LC alterations have also been reported in aging and neurodegenerative conditions, with studies in PD and MCI showing greater disruptions of left‐hemisphere LC functional connectivity.^56^, ^57^ Moreover, increased left‐sided LC contrast has been associated with aging and PD.^12^, ^55^ However, no clear explanation was provided for this LC lateralization effect. Our findings offer a potential interpretation, suggesting that left LC connections are stronger than the right ones. Thus, the LC can be included among human brain structures with left‐hemisphere dominance.^78^ We found that the stronger left LC connections were not only reproducible but also functionally meaningful, as they were more affected in both aging and AD and were more strongly associated with tau pathology accumulation in the entorhinal cortex.

At the molecular level, the additional rostral LC connectivity loss we found in AD was associated with transcriptomic profiles related to synaptic function, neuron projection, and the AD–presenilin pathway. The LC‐norepinephrine system supports synaptic function and plasticity,^79^ and its disruption can lead to synaptic collapse,^80^ a hallmark of AD. LC signaling also promotes adaptive myelination^81^ and facilitates microglial clearance of amyloid plaques in transgenic mice with mutant presenilin‐1, consistent with the enrichment of the AD‐presenilin pathway identified in our analyses.^82^ Together, these findings suggest that the additional loss of rostral LC connectivity in AD is closely linked to molecular pathways critical for brain resilience against AD pathology.

Notably, the cortical regions affected by LC connectivity loss in AD included areas involved in early AD pathology, particularly the entorhinal cortex.^24^ While debate continues about whether tau pathology begins in the LC or the entorhinal cortex,^68^, ^83^, ^84^ our results suggest that LC‐entorhinal connectivity may drive early tau accumulation as LC gradients were better predictors of entorhinal tau burden than the reverse relationship. However, future studies using PET tracers sensitive to pre‐tangle tau pathology and without off‐target binding to neuromelanin in the LC are needed to address this debate.

We also observed a striking pattern: correlations between LC connectivity gradients and entorhinal tau pathology were in opposite directions in cognitively normal Aβ+ (CN Aβ+) and AD Aβ+ individuals, with no significant associations found in the MCI Aβ+ group. This pattern is in line with previous studies showing that, in early AD, functional connectivity may increase due to amyloid‐driven hyperexcitability, which diminishes during MCI and disappears in dementia stages.^85^ On the other hand, there is ongoing debate about whether these early connectivity increases may serve as a compensatory mechanism that maintains cognitive function despite accumulating pathology.^2^, ^86^, ^87^ Our novel LC gap measure reinforces this later interpretation: individuals with a more preserved gradient (negative LC gap) showed slower cognitive decline and tau accumulation over time. These results align with animal studies showing that preserved LC function can reduce tau‐related neurodegeneration^88^ and neuropathological studies showing that greater LC neuron density is associated with slower cognitive decline and lower tau burden.^11^, ^58^, ^59^ They also complement emerging evidence that brain connectivity and cognitive reserve can mitigate the clinical impact of AD pathology.^89^

In line with the LC's role as a central neuromodulatory hub promoting beneficial neuronal adaptations,^7^ we found that LC gradient parameters were associated with cognitive, sleep, motor, and psychiatric functions in the aging cohort, highlighting its broad influence on brain functions and behavior, and in line with previous reports of a link between the LC and memory, attention and anxiety.^3^, ^90^ Additionally, we found a relation between LC and emotional memory aligning with evidence indicating that the LC is involved in emotional memory processing through its influence on sleep, crucial for memory consolidation.^91^ This association is particularly significant given the importance of sleep in the pathogenesis of AD.^92^ Furthermore, LC connectivity within the AD cohort was also linked to preclinical cognitive and memory measures commonly used in clinical assessments. These results further support the role of the LC in modulating cognition across both healthy aging and disease. Interestingly, LC parameters associated with the neuropsychological tests differed between cohorts: stronger associations were observed with complex cubic connectivity features in aging and simpler linear and quadratic parameters in AD. This difference could reflect that, in healthy aging, subtle changes may only be captured by complex metrics, while in AD—where neurodegeneration is more pronounced—connectivity alterations are easily detected with basic measures.

Our study has some limitations that need to be taken into consideration. First, the relatively small sample size of the AD and the cross‐sectional data from the two independent cohorts may limit the statistical power and prevent capturing the progression of LC connectivity changes over time. Future studies should aim to replicate these findings using longitudinal and larger datasets. Although it was beyond the scope of the current study, the absence of in vivo tau pathology measurements within the LC, as well as assessments of other relevant pathologies, limits our ability to identify the primary drivers of the observed connectivity changes. Furthermore, the dorsal‑predominant structural loss shown in our results might affect our ability to differentiate dorsal and ventral LC projections in MNI‑normalized space. It is not possible to determine whether a weakened dorsal–ventral gradient reflects de‐differentiation due to structural loss or adaptive reorganization of projections, although previous LC pathology literature in aging and AD^93^ supports the de‐differentiation interpretation. Finally, our transcriptomic analysis has inherent limitations: lacking individual‐level data, we relied on transcriptomic data derived from six *post‐mortem* brains to interpret the differences in LC connectivity between aging and AD. Although this approach has been successfully used in previous studies and is based on the premise that spatial variation in gene expression across brain regions exceeds inter‐individual variability,^47^ the findings presented here should be viewed as preliminary. However, obtaining such data from real individuals in a cohort of this scale is nearly unfeasible; therefore, although this represents a clear limitation, it remains the most viable approach currently available.

Despite these limitations, our findings revealed a distinct organization of LC anatomical connections, following a dorsal–ventral gradient with marked asymmetry. This pattern was consistently observed across two independent cohorts, even though the brain scans were acquired using different imaging sequences—suggesting that the gradient structure is stable across methodological variations. We identified key changes in LC connectivity linked to both aging and AD, and found associations with behavioral measures, underlying pathology, and gene expression profiles. Notably, individuals with slower disease progression who showed a specific LC connectivity pattern, preserving the dorsal projections, also exhibited slower tau accumulation over time, pointing to a possible protective role of this organization. These results emphasize the value of studying the connectivity in neuromodulatory structures, aligning with a recent perspective that highlight their relevance in the understanding of AD pathophysiology and their potential contribution to resilience mechanisms in AD,^94^ as well uncovering the mechanisms that separate healthy aging from disease.

## CONFLICT OF INTEREST STATEMENT

All authors report no conflicts of interest. The authors have no potential conflicts of interest to disclose. Author disclosures are available in the Supporting Information.

## CONSENT STATEMENT

In the Cam‐CAN dataset, written informed consent was provided by all subjects after the study received ethical approval from the Cambridgeshire 2 Research Ethics Committee (reference: 10/H0308/50) and the adherence to the Helsinki Declaration was verified. In the ADNI cohort, written informed consent was obtained from all participants or their representatives, with ethical approval granted at each participating ADNI site.

## Supporting information




**Supporting Information**: alz71737‐sup‐0001‐SuppMat.docx

**Supporting Information**: alz71737‐sup‐0002‐SuppMat.pdf

## Data Availability

The data used in this study were obtained from the Cam‐CAN cohort^25^ (https://www.cam‐can.org/) and the ADNI database (adni.loni.usc.edu). The Cam‐CAN's imaging and behavioral data are publicly accessible at https://camcan‐archive.mrccbu.cam.ac.uk/dataaccess/. The investigators within the ADNI contributed to the design and implementation of ADNI and/or provided data but did not participate in analysis or writing of this report. A complete listing of ADNI investigators can be found at: http://adni.loni.usc.edu/wp‐content/uploads/how_to_apply/ADNI_Acknowledgement_List.pdf
