# Supplementary material for: Vulnerability of locus coeruleus connections to aging and Alzheimer's disease
Source: Alzheimers Dement. 2026 Aug 12;22(8):e71737. doi: 10.1002/alz.71737 (PMC13463321; doi:10.1002/alz.71737)
Supplement: Supplementary file 2 [file ALZ-22-e71737-s001.docx]

**Supplementary Materials for**

**Vulnerability of locus coeruleus connections to aging and Alzheimer’s Disease**

Zufiria-Gerbolés *et al*.

*Corresponding authors:

Blanca Zufiria Gerbolés ([blanca.zufiria.gerboles@ki.se](mailto:blanca.zufiria.gerboles@ki.se)) // Joana B. Pereira ([joana.pereira@ki.se](mailto:joana.pereira@ki.se))

This file includes:

**Supplementary Analyses**

Connectivity gradients outperform anatomical connections in predicting aging and cognition.

**Supplementary Tables**

Supplementary Table 1: Spearman partial correlations while correcting for age, sex and education between the LC gradient parameters and age in the Cam-CAN cohort.

Supplementary Table 2: Kruskal-Wallis tests were conducted, followed by post hoc pairwise comparisons, to compare TSM parameters describing the LC gradients across the AD continuum in the ADNI cohort.

Supplementary Table 3: Summary of Spearman partial correlations (correcting for age, sex and education) between LC Gradient Parameters and Tau Pathology in the AD continuum cohort.

**Supplementary Figures**

Supplementary Figure 1: Distribution of participants across age bins (Cam‑CAN cohort) and cognitive/amyloid-pathology groups (ADNI).

Supplementary Figure 2: Brain‑wide LC connectivity gradients and dimensionality analyses.

Supplementary Figure 3: LC projections to the rest of the brain in the aging (Cam-CAN) and AD continuum (ADNI) cohorts

Supplementary Figure 4: Reproducibility of LC gradient correspondence assessed via bootstrapping.

Supplementary Figure 5: Breakpoint detection in significant partial correlations (correcting for age, sex and education) between LC gradient parameters and age in the Cam-CAN cohort

*Supplementary Figure 6*: Association between LC connectivity differences between aging and AD and gene expression profiles

*Supplementary Figure 7*: Comparison, using linear regression models ( *P < 0.05, **P < 0.01, ***P < 0.001), of predictive performances of LC gradients and entorhinal tau for longitudinal cognitive decline

**Connectivity Gradients Outperform Anatomical Connections in Predicting Aging and Cognition**

To assess whether connectivity gradients provided additional value beyond the individual anatomical connections assessed using fiber numbers, we conducted an additional analysis comparing the ability of both methods to predict age in the Cam-CAN cohort and a general cognitive test in the ADNI cohort (mPACCtrailsB). We fitted a third-order trend surface model (TSM) [41] to the gradients to described them in the space with 9 coefficients across left-right (*x*), dorsal-ventral (*y*) and rostral-caudal (z) directions, which were either linear (*x*, *y*, *z*), quadratic (*x²*, *y²*, *z²*) or cubic (*x³*, *y³*, *z³*). Using partial least squares regression (PLSR), we found that, compared to the standalone connections, the gradient parameters were superior in predicting aging (R²gradients = 0.67 > R²projections = 0.38) as well as cognition (R²gradients = 0.27 > R²projections = 0.26). These results show that the gradients provide additional information beyond traditional connectivity measures.

| **x** | **y** | **z** |
| --- | --- | --- |
| rho = 0.028, p=0.816 | rho = -0.213, P = 0.080 | **rho = 0.495, P < 0.001** |
| **x^2^** | **y^2^** | **z^2^** |
| **rho =-0.379, P = 0.0014** | rho = 0.104, P = 0.395 | **rho = 0.480, P < 0.001** |
| **x^3^** | **y^3^** | **z^3^** |
| rho = -0.002, P =0.985 | rho = -0.009, P =0.940 | **rho = -0.495, P < 0.001** |

**Supplementary Table 1**. **Spearman partial correlations (rho) followed by p-values (P) while correcting for age, sex and education between the LC gradient parameters and age in the Cam-CAN cohort.** Significant results (P_FDR_<0.01) are highlighted in bold, In the lateral direction, parameter x^2^ showed a significant negative correlation with age (rho = -0.38, p = 0.001). In the rostro-caudal direction, we observed significant correlations for z (rho = 0.5 P < 0.00p), z^2^ (rho = 0.48 P < 0.001), and z^3^ (rho = -0.5, p = P < 0.001), reflecting structural changes along this axis.

| **Statistical tests** | | **x** | **y** | **z** |
| --- | --- | --- | --- | --- |
| **Kruskal-Wallis**  **(Chi-squared and p-value)** | | ***103.39***  ***(P < 0.001)*** | ***40.31***  ***(P < 0.001)*** | ***13.11***  ***(P=0.0014)*** |
| **Pairwise comparison**  **(Mean Difference and p-value)** | **CN vs MCI** | *-22.28*  *(P=0.16)* | ***60.42***  ***(P < 0.001)*** | ***-44.42***  ***(P < 0.001)*** |
|  | **CN vs AD** | ***-117.43 (P < 0.001)*** | ***72.78***  ***(P < 0.001)*** | *-22.18*  *(P=0.16)* |
|  | **MCI vs AD** | ***-95.15***  ***(P < 0.001)*** | *-16*  *(P=0.57)* | *22.24*  *(P=0.16)* |
|  |  | **x^2^** | **y^2^** | **z^2^** |
| **Kruskal-Wallis**  **(Chi-squared and p-value)** | | ***11.81***  ***(P=0.0027)*** | ***68.49***  ***(P < 0.001)*** | ***58.64***  ***(P < 0.001)*** |
| **Pairwise comparison**  **(Mean Difference and p-value)** | **CN vs MCI** | *6.39*  *(P=0.86)* | *-11.18*  *(P=0.63)* | *-20.88*  *(P=0.2)* |
|  | **CN vs AD** | *-32.89*  *(P=0.02)* | ***81.8***  ***(P < 0.001)*** | ***-89.76***  ***(P < 0.001)*** |
|  | **MCI vs AD** | *-39.29*  *(P=0.003)* | ***-92.98***  ***(P < 0.001)*** | ***-68.88***  ***(P < 0.001)*** |
|  |  | **x^3^** | **y^3^** | **z^3^** |
| **Kruskal-Wallis**  **(Chi-squared and p-value)** | | ***115.85***  ***(P < 0.001)*** | ***57.9***  ***(P < 0.001)*** | ***77.32***  ***(P < 0.001)*** |
| **Pairwise comparison**  **(Mean Difference and p-value)** | **CN vs MCI** | *-6.22*  *(P=0.86)* | *-23.34*  *(P=0.13)* | ***50.19***  ***(P < 0.001)*** |
|  | **CN vs AD** | ***111.11***  ***(P < 0.001)*** | ***-89.94***  ***(P < 0.001)*** | ***-57.6***  ***(P < 0.001)*** |
|  | **MCI vs AD** | ***117.34***  ***(P < 0.001)*** | ***-37.84***  ***(P < 0.001)*** | ***-107.79***  ***(P < 0.001)*** |

**Supplementary Table 2.** **Kruskal-Wallis tests were conducted, followed by post hoc pairwise comparisons, to compare TSM parameters describing the LC gradients across the AD continuum in the ADNI cohort.** Results (P_FDR_<0.01) showed that early dementia (AD) differs from mild cognitive impairment (MCI) and cognitively normal (CN) for parameters in the three directions while CN and MCI can be best differentiated by the ventro-dorsal parameter y and rostro-caudal parameters z and z^3^.

| **Gradient parameters** | **CN (Aβ+)** | **MCI (Aβ+)** | **AD (Aβ+)** |
| --- | --- | --- | --- |
| x | **0.359 (P < 0.001)** | 0.011 (P=0.9126) | 0.131 (P=0,2005) |
| y | **0.320 (P=0.0014)** | 0.245 (P=0.0154) | 0.083 (p=0.4208) |
| z | **-0.310 (P=0.0020)** | -0.188 (P=0. 0655) | 0.059 (P=0.5644) |
| x^2^ | **0.359 (P < 0.001)** | -0.106 (P=0,2995) | 0.005 (P=0.9648) |
| y^2^ | -0.070 (P=0.4927) | -0.067 (P=0.5126) | -0.144 (P=0.1607) |
| z^2^ | **-0.466 (P < 0.001)** | 0.157 (P=0.1257) | **0.502 (P < 0.001)** |
| x^3^ | -0.175 (P=0.0856) | 0.072 (P=0.4819) | -0.117 (P=0.2548) |
| y^3^ | -0.160 (P=0.1169) | -0.150 (P=0.1425) | -0.173 (P=0.0900) |
| z^3^ | **0.300 (P=0.0028)** | 0.101 (P=0,3249) | 0.118 (P=0.2510) |

**Supplementary Table 3. Summary of Spearman partial correlations (correcting for age, sex and education) between LC Gradient Parameters and Tau Pathology in the AD continuum cohort**. This table presents the significant associations identified between tau pathology and various LC gradient parameters in different groups of Aβ+ individuals. Tau pathology was measured at baseline with the standard uptake value ratio (SUVR) from positron emission tomography (PET) imaging. In cognitively normal (CN) Aβ+ individuals, significant correlations were observed with parameters x, y, z, x^2^, z^2^, and z^3^. In Alzheimer's disease (AD) Aβ+ individuals, a significant correlation was found specifically with the z^2^ parameter. Notably, the direction of the correlation between the z^2^ gradient parameter and Tau-PET SUVR in the entorhinal cortex differed between CN Aβ+ and AD Aβ+ individuals, with a negative correlation in CN Aβ+ (rho = -0.46, p = 1.5 × 10⁻⁶) and a positive correlation in AD Aβ+ (rho = 0.5, p = 1.6 × 10⁻⁷). No significant correlations were found for mild cognitive impaired (MCI) Aβ+ individuals.

**Supplementary Figure 1. Distribution of participants across age bins (Cam‑CAN cohort) and cognitive/amyloid-pathology groups (ADNI).** a) Age distribution of the Cam‑CAN cohort divided into 71 one‑year age bins ranging from 18 to 88 years old. The plot displays the number of participants in each bin. As expected for a population-based sample, the distribution closely follows a Gaussian-like pattern, with a higher concentration of participants in mid-life and fewer individuals at the younger and older extremes; with some imbalance between specific age groups, particularly in age bin 21 and age bin 79, where representation appears uneven. b) Distribution of participants in the ADNI cohort, grouped according to amyloid‑β status and cognitive diagnosis: cognitively normal Aβ− (CN Aβ−, n = 77), cognitively normal Aβ+ (CN Aβ+, n = 16), mild cognitive impairment Aβ+ (MCI Aβ+, n = 31), and Alzheimer’s disease Aβ+ (AD Aβ+, n = 8). Group sizes vary substantially, reflecting differences in prevalence and recruitment across diagnostic categories.

**Supplementary Figure 2. Brain‑wide LC connectivity gradients and dimensionality analyses.** a) Whole‑brain map of the dominant structural connectivity gradient of the locus coeruleus (LC) for the cognitively normal (CN) older subgroup (age 55–86 years) in the aging cohort. Colors indicate the cortical regions most strongly correlated with each LC voxel. b) Intrinsic dimensionality analysis for the CN older subgroup in the aging cohort. Plots show the hypersphere radius and root‑mean‑square deviation (RMSD) as a function of embedding dimensionality. This pattern indicates that the LC similarity matrices are effectively low-dimensional and largely dominated by a single principal axis of variation, demonstrating that the first (dominant) gradient captures most of the explained variance (25% of the total variance in ADNI and 24% in Cam-CAN). c) Whole‑brain map of the dominant LC connectivity gradient for the CN older subgroup in the AD continuum cohort (same visualization procedure as in panel a). d) Corresponding intrinsic dimensionality analysis for the AD continuum cohort. As in panel b, the hypersphere and RMSD curves demonstrate a steep drop from dimension 1 to 2 but negligible gains thereafter, confirming that the LC connectivity structure is effectively low‑dimensional and dominated by a single primary gradient.

**Supplementary Figure 3. LC projections to the rest of the brain in the aging (Cam-CAN) and AD continuum (ADNI) cohorts**. All participants underwent diffusion weighted imaging (DWI) to identify the anatomical connections projecting from the LC to other cortical and subcortical brain areas of the Brainnetome atlas. For the two cohorts, a) Cam-CAN cohort and b) ADNI cohort, participants with matched age (55-86 years old) and cognitive normal (CN) were included. In both cohorts, stronger connections project to same regions the left hemisphere. These maps showed a high degree of overlap between the two cohorts (R = 0.8823, p < 0.001).

**Supplementary Figure 4. Reproducibility of LC gradient correspondence assessed via bootstrapping.** To evaluate the robustness of the gradient correspondence between cohorts, we performed a bootstrapping procedure in which 20 participants were randomly selected from each group (with/without replacement) and LC gradients were recomputed at each iteration (see Methods: Computation of LC gradients). This procedure was repeated 100 times, and the correlation between gradients derived from the two cohorts was calculated for each iteration. The distribution of correlation coefficients demonstrated high reproducibility, with a mean correlation of 0.87 ± 0.0055.

**Supplementary Figure 5. Breakpoint detection in significant partial correlations (correcting for age, sex and education) between LC gradient parameters and age in the Cam-CAN cohort**. To identify the point at which the significant correlation between the two variables becomes more pronounced, we applied a rolling correlation analysis with derivative-based breakpoint detection. A moving window correlation (window size = 15 years) was computed to capture local variations in correlation strength. The first derivative of the rolling correlation quantified the rate of change, while the second derivative identified the point of maximum variation in the correlation. The breakpoint was defined as the age where the second derivative reached its minimum or maximum value (depending on the sign of the correlation), indicating a significant shift in the correlation pattern. Breakpoint ages at which correlation accelerates are a) 46 for parameter z, b) 52 for parameter x2, c) 50 for parameter z2 and d) 51 for parameter z3

**Supplementary Figure 6.** **Association between LC connectivity differences between aging and AD and gene expression profiles**. a) Estimated mean square prediction error for the first 20 components from the partial least squares (PLS) regression model. b) Explained variance for the first 20 components from the PLS. c) First PLS component (PLS1) scores plotted on a brain surface. d) Spearman partial correlation (rho) and p-value (P) between PLS1 scores and LC differences (between aging and AD) in modular variability (each point is a brain region).

**Supplementary Figure 7. Comparison, using linear regression models (*P < 0.05, **P < 0.01, ***P < 0.001), of predictive performances of LC gradient parameters and entorhinal tau for longitudinal cognitive decline** in a) cognitively normal (CN) Amyloid-positive (Aβ+), c) mild cognitive impaired (MCI) Aβ+, and d) early dementia (AD) Aβ+ subjects. LC gradient parameters are superior predictors in CN Aβ+, while entorhinal tau is more effective in MCI Aβ+ and AD Aβ+. b) Predicting entorhinal tau from the LC gradient parameters is more effective than predicting the LC gradient parameters from entorhinal tau in CN Aβ+ participants. These findings suggest that the spread of the pathology would extend from the LC to the entorhinal cortex.
